# *Chlamydia trachomatis* infection and risk of ovarian cancer: a systematic review and meta-analysis

**DOI:** 10.1590/S1678-9946202567034

**Published:** 2025-06-09

**Authors:** Pei Wang, Xiuxiu You, Xianjing Zeng, Qingmei Peng

**Affiliations:** 1 Xianyang Central Hospital in Shaanxi Province, Department of Gynecology and Obstetrics, Xianyang, Popular Republic of China; 2 The Affiliated Hospital of Southwest Medical University, Department of Gynecology, Luzhou, Popular Republic of China; 3 Affiliated Hospital of Jinggangshan University, Department of General Practice Medicine, Ji'an, Popular Republic of China; 4 Jinggangshan University, Clinical School of Medicine, Department of Obstetrics and Gynecology, Ji'an, Popular Republic of China

**Keywords:** Chlamydia trachomatis, Infection, Ovarian cancer, Systematic review, Meta-analysis

## Abstract

*Chlamydia trachomatis* infection has been implicated as a potential risk factor for ovarian cancer (OC), but evidence remains inconclusive. This study aims to assess the association between *C. trachomatis* infection and OC risk using a systematic review and meta-analysis. A comprehensive literature search was conducted in PubMed, Embase, Scopus, Web of Science, and SciELO from their inception to October 2024. Observational studies examining the association between *C. trachomatis* infection and OC risk were included. Pooled odds ratios (ORs) were calculated using random-effects models. Subgroup and sensitivity analyses were performed based on diagnostic methods and control group types, and publication bias was assessed using Egger's test. Out of 3,288 records, 11 studies involving 4,518 participants were included. The overall meta-analysis revealed a non-significant association between *C. trachomatis* infection and OC risk (OR = 1.30, 95% CI = 0.99–1.70). However, sensitivity analysis excluding two studies demonstrated a significant association (OR = 1.37, 95% CI = 1.16–1.61). Subgroup analysis showed that PCR-diagnosed *C. trachomatis* infection was significantly associated with an increased risk (OR = 6.64, 95% CI = 1.62–25.71), while serology-based studies yielded non-significant results. Heterogeneity was high (*I*² = 70.83%), and publication bias was detected (Egger's test p = 0.015). These findings highlight the role of infection chronicity in OC pathogenesis and suggest that diagnostic methodology significantly impacts observed associations. Future research should employ longitudinal designs with serial molecular testing to establish temporality and evaluate whether targeted *Chlamydia* screening or treatment interventions could mitigate OC risk in high-prevalence populations.

## INTRODUCTION

Ovarian cancer (OC) is one of the most common gynecological cancers and ranks eighth in cancer-related mortality among women^1^. In 2020, it was estimated that ~314,000 new cases of OC were diagnosed globally, with over 207,000 deaths attributed to the disease^2^. OC often presents at advanced stages due to lack of early symptoms and effective screening methods, leading to poor survival rates^3^. Risk factors for OC include advanced age, family history of ovarian or breast cancer, inherited mutations in BRCA1/2 genes, reproductive history, and lifestyle factors such as diet and obesity^3^. Despite these established risk factors, a growing body of evidence suggests that infections, particularly sexually transmitted infections (STIs) such as *Chlamydia trachomatis*, may contribute to the development of ovarian cancer^4,5^. Infections can lead to chronic inflammation, which has been identified as a contributing factor to carcinogenesis^6^. Chronic pelvic inflammatory disease (PID), a common consequence of untreated *C. trachomatis* infections, has been associated with an increased OC risk^7^. Studies suggest that women with a history of STIs may have up to a twofold higher risk of developing OC compared to those without such a history^4^. Furthermore, *C. trachomatis*, the most common bacterial STI worldwide, has been shown to induce genetic alterations, persistent inflammation, and immune evasion, all of which are mechanisms contributing to cancer development^8^.

*Chlamydia trachomatis* is a highly prevalent bacterial STI, with an estimated 129 million new cases reported annually worldwide^9^. The infection is most common among young adults, particularly women aged 15–24 years, and its burden is disproportionately higher in low- and middle-income countries^10^. Despite being curable with antibiotics, chlamydial infection is often asymptomatic, leading to delayed diagnosis and increased risk of complications such as PID, ectopic pregnancy, and infertility^11^. The pathophysiology of *C. trachomatis* in OC involves chronic inflammation, which is a well-established risk factor for cancer. When left untreated, *C. trachomatis* can cause persistent infection in the fallopian tubes and ovaries, leading to scarring, chronic inflammation, and genetic alterations in the epithelial cells. This inflammatory microenvironment facilitates DNA damage, inhibits normal apoptotic mechanisms, and promotes cellular proliferation, ultimately increasing the risk of malignant transformation^12^.

While various studies have explored the association between *C. trachomatis* and ovarian cancer^13^, significant research gaps remain. Many studies have been limited by small sample sizes, varying populations, geographic regions, and diagnostic methods, leading to inconsistent results. There is a critical need for comprehensive, high-quality meta-analyses to better assess this association and understand the underlying biological mechanisms. Therefore, our study aims to conduct a systematic review and meta-analysis to synthesize existing data on the link between *C. trachomatis* infection and OC risk. By addressing these gaps, we hope to clarify the potential role of *C. trachomatis* as a modifiable risk factor, which could have important implications for disease prevention and creation of public health policies.

## MATERIALS AND METHODS

### Study design, search strategy, and study selection

This study follows the Preferred Reporting Items for Systematic Reviews and Meta-Analyses (PRISMA) guidelines. A comprehensive literature search was performed using the electronic databases PubMed, Embase, Scopus, Web of Science, and SciELO. The first 20 pages of Google Scholar were also searched. The search covered studies published from inception until October 1, 2024. The following key terms and their combinations were used: "*Chlamydia trachomatis*," "bacterial infection," "ovarian cancer," "ovarian neoplasm," "ovarian tumors," "sexually transmitted infections," and "risk factors." Boolean operators (AND, OR) were applied to refine search results. No language restrictions were employed. Additionally, the reference lists of relevant articles and reviews were manually searched to identify any studies that might have been overlooked during the database search.

Studies were included if they met the following criteria: (1) investigated the association between *C. trachomatis* infection and OC risk; (2) provided original, peer-reviewed data from observational studies (e.g., cohort, case-control, or cross-sectional designs); (3) reported odds ratios (ORs), relative risks (RRs), hazard ratios (HRs), or provided sufficient data to calculate these risk estimates. Studies were excluded if they (1) did not include an appropriate control group; (2) lacked adequate data to calculate effect estimates; (4) had overlapped data with other studies; (4) review articles, case reports, editorials, or conference abstracts.

### Data extraction

Two independent reviewers screened all titles and abstracts for eligibility. If they met the inclusion criteria, the full text was reviewed. Discrepancies were resolved by discussion or consultation with a third reviewer. The following information was extracted from each study: study characteristics (author names, year of publication, country, and study design); population characteristics (sample size, participant demographics, and geographic region); exposure and outcome measures (method of *Chlamydia trachomatis* infection diagnosis [serological, PCR, etc.], ovarian cancer diagnosis [histologically or clinically confirmed], and confounding variables); and effect estimates (reported ORs, RRs, or HRs with 95% confidence intervals [CIs], and adjustments made for confounding factors).

### Quality assessment

The quality of the included studies was assessed using the Newcastle-Ottawa Scale (NOS) for observational studies^14^. Studies were evaluated based on three main domains: selection of study groups, comparability of groups, and ascertainment of outcomes. Studies scoring 7 points or higher were considered high-quality, while those scoring less than 7 points were considered to have a higher risk of bias.

### Statistical analysis

A random-effects meta-analysis was performed to pool the effect estimates across studies^15^, accounting for between-study heterogeneity. The primary outcome was the association between *C. trachomatis* infection and OC risk, expressed as a pooled odds ratio (OR) with 95% CIs. Heterogeneity among studies was assessed using the *I* ^2^ statistic, with values above 50% indicating substantial heterogeneity^16^. Subgroup analyses was performed based on diagnostic methods for *C. trachomatis* and type of control group. Sensitivity analyses were conducted by excluding studies to test the robustness of the findings. Cumulative meta-analyses were applied to determine the reliability of the estimated ORs. Publication bias was evaluated using funnel plots and Egger's test^17^. All statistical analyses were performed using the Stata software version 17 (STATA Corp., College Station, TX, USA). P-values less than 0.05 were considered statistically significant.

## RESULTS

### Study selection and characteristics

**Figure 1:**
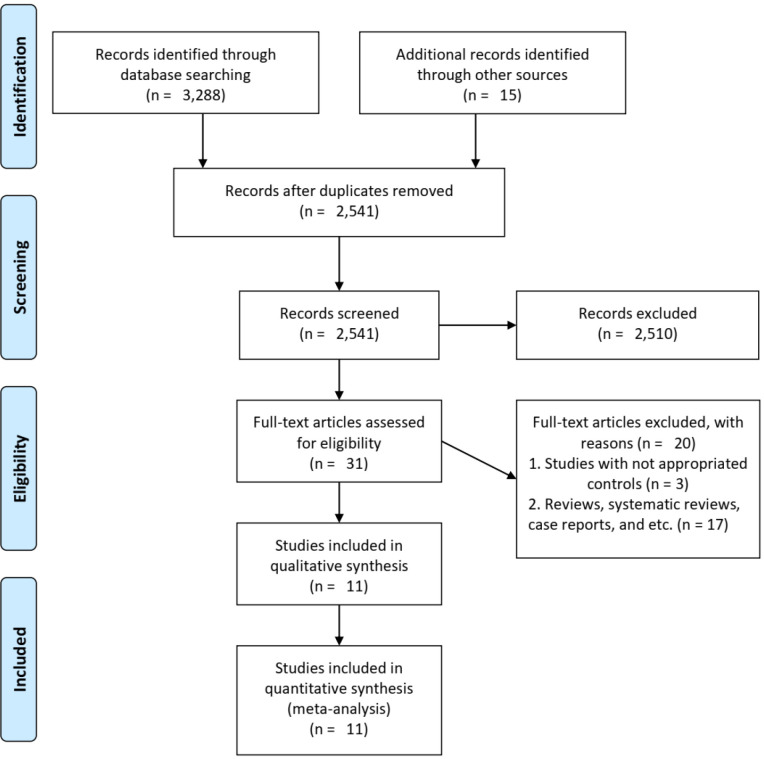
PRISMA flow diagram illustrating the study selection process.

A total of 3,288 records were identified after database searching, with 15 additional records sourced from other sources. After removing duplicates, 2,541 records were screened based on titles and abstracts. Of these, 2,510 records were excluded during title/abstract screening, primarily comprising: (1) non-human or in vitro studies (n = 1,203); (2) studies of non-ovarian cancers (n = 692); (3) articles focusing solely on *Chlamydia pathogenesis* without cancer outcomes (n = 415), and (4) duplicate publications or conference abstracts without full data (n = 200). In total, 31 full-text articles were assessed for eligibility. Of these, 20 were excluded for various reasons: three studies had inappropriate comparison groups (e.g., combining benign and malignant ovarian cancer) or failed to recruit control groups, while the remaining 17 studies contained non-original or non-analyzable data, including letters to the editor, correspondences, reviews, systematic reviews, case reports, and case series. Ultimately, 11 studies^4,5,18–25^ were included in both the qualitative and quantitative synthesis (Figure 1). The included studies comprised diverse geographical regions and study designs, as summarized in Table 1. These studies were conducted from 1983 to 2023 in the USA, Sweden, Iran, Egypt, Canada, Finland, and Poland. Most studies employed nested case-control design; two studies had population-based case-control design, one study has hospital-based case-control design, and one study had comparative cross-sectional design.

**Table 1:** Main characteristics of studies included.

Study	Study period	Country	Study design	Diagnostic methods	Type of screened antibody in serological methods	Mean ages or age range		Patients with ovarian cancer		Controls		Quality score
						Cases	Controls	Number	*Chlamydia* positive	Number	*Chlamydia* positive	
Dadashi *et al*.^18^	2014-2015	Iran	HBCC	Standard PCR	-	NS	NS	62	14	62	0	Moderate
Fortner *et al*.^4^	2015-2016	USA	NCC	Serology (MFIA)	Antibodies against Pgp3	34-81	35-80	337	66	337	40	High
Idahl *et al*.^19^	1994-2001	Sweden	NCC	PCR (Gen-Probe TMA)	-	58.9	55.9	52	0	134	0	High
Idahl *et al*.^20^	1993-2001	Sweden	NCC	Serology (MIF)	*C. trachomatis* specific IgG	31-82	18-87	57	15	386	89	High
Idahl *et al*.^25^	1992-2000	Sweden	NCC	Serology (MFIA)	Antibodies against cHSP60-1	29.9-80.7	30.1-79.3	791	144	1669	243	High
Laban *et al*.^21^	2008-2017	Egypt	CCS	qRT-PCR	-	54.8	58.1	55	25	12	2	High
Ness *et al*.^22^	1993-1999	Canada	PBCC	Serology (ELISA)	IgG anti-EBs (*C. trachomatis* serovar D)	18-84	18-84	117	32	171	27	High
Ness *et al*.^23^	2003-2006	USA	PBCC	Serology (ELISA)	IgG anti-EBs (*C. trachomatis* serovar D)	25-63	24-84	521	62	766	138	High
Skarga *et al*.^5^	1983-2016	Finland	NCC	Serology (MFIA)	Antibodies against Pgp3 and cHSP60	15-45	16-45	484	227	484	220	High
Trabert *et al*.^24^	2001-2003	Poland	NCC	Serology (MFIA)	Antibodies against ≥3 of: MOMP-A, MOMP-D, MOMP-L2, Tarp-F1, Tarp-F2, HSP60-1	55.5	55.6	244	89	556	158	High
Trabert *et al*.^24^	2001-2003	USA	NCC	Serology (MFIA)	Antibodies against ≥3 of: MOMP-A, MOMP-D, MOMP-L2, Tarp-F1, Tarp-F2, HSP60-1	63.3	63.1	160	37	159	34	High

HBCC = Hospital-based case-control; NCC= Nested case-control; CCS = Comparative cross-sectional; PBCC = Population-based case-control; PCR = Polymerase chain reaction; NS = not specified; EB = elementary bodies; MFIA = Multiplex fluorescent immunoassay; MIF = Microimmunofluorescence; TMA = Transcription-mediated amplification; qRT-PCR = Quantitative reverse transcription PCR.

All studies enrolled patients with histopathological-confirmed epithelial ovarian cancer (EOC), with diagnoses based on tissue biopsy or surgical specimen analysis. For *C. trachomatis* infection, detection methods included either serological assays targeting antibodies against different bacterial antigens or molecular techniques identifying bacterial DNA. Among the included studies, three used molecular assays (PCR, gene-probe TMA, and qRT-PCR), while eight employed serology-based methods (MFIA in five, ELISA in two, and MIF in one). Overall, most studies were of high methodological quality, as assessed by the NOS, with one study rated as moderate. The sample sizes of OC cases ranged from 52 to 791, while control group sizes varied from 12 to 1,669. The mean age or age range of participants also varied significantly, with studies including participants aged from 18 to 87 years. Most studies reported a higher prevalence of *C. trachomatis* positivity among OC cases compared to controls.

Among the included studies, only two studies stratified their data by EOS histological subtypes (serous, clear cell, endometrioid, and mucinous). In the study by Idhal *et al*.^25^, CT infection was significantly associated with EOC based on antibodies against cHSP60-1 (RR = 1.36; 95% CI: 1.13–1.64). Subgroup analysis revealed this association was significant for the serous subtype (RR = 1.44; 95% CI: 1.12–1.85) but not for other subtypes. Notably, while antibodies against Pgp3 showed no significant association with overall EOC or serous, clear cell, and endometrioid subtypes, a significant association was observed for the mucinous subtype (RR = 2.30; 95% CI: 1.22–4.32). Moreover, in the study by Skarga *et al*.^5^, a non-significant association was found between CT infection and EOC and all histological subtypes.

### Synthesis of results

**Figure 2:**
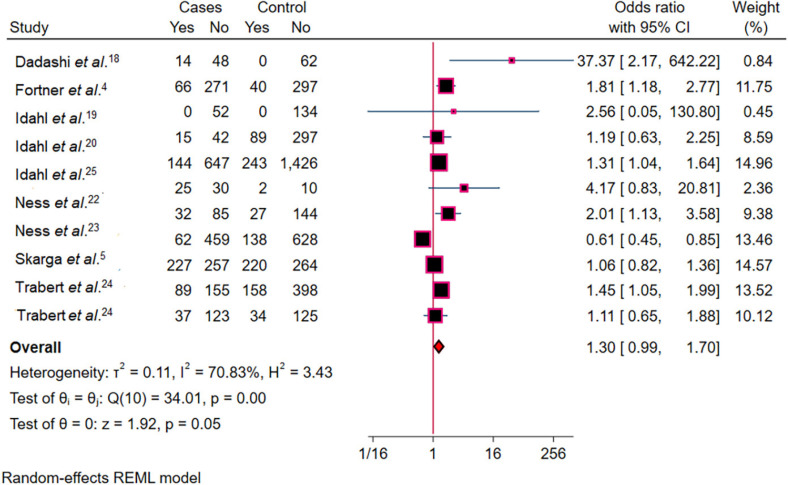
Forest plot showing the pooled random-effects analysis of the association between *Chlamydia trachomatis* infection and ovarian cancer, with OR and 95% CI.

**Figure 3:**
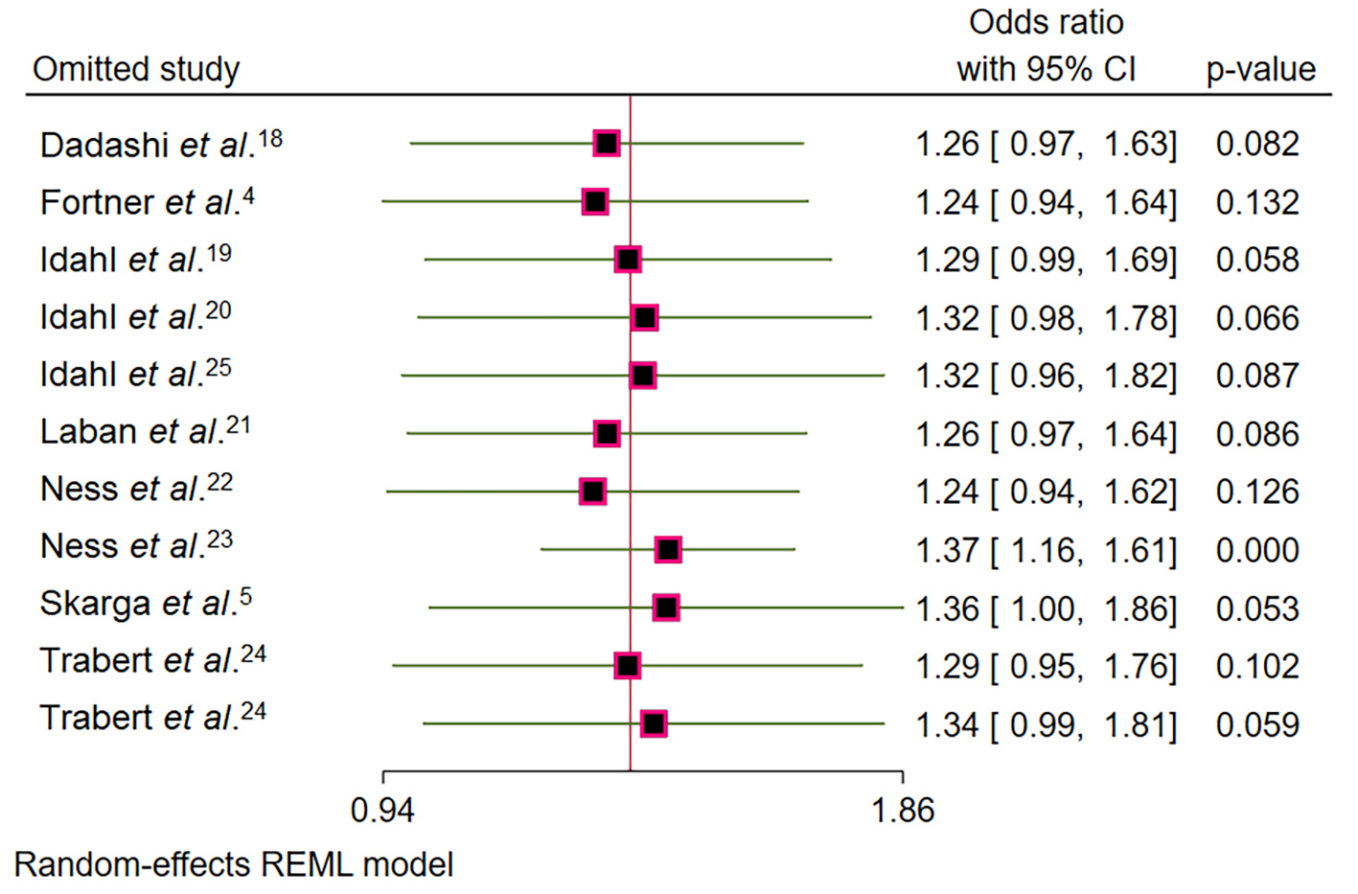
Sensitivity analysis assessing the impact of individual studies on the overall association between *Chlamydia trachomatis* infection and ovarian cancer. The plot shows how the pooled OR changes when each study is excluded, with 95% CI for the recalculated estimates.

The random-effects model metanalysis revealed a non-significant association between *C. trachomatis* infection and increased risk of OC (OR = 1.30, 95% CI = 0.99–1.70; Figure 2). However, in sensitivity analyses, when studies by Ness *et al*.^23^ and Skarga *et al*.^5^ were excluded, the association became statistically significant (OR = 1.37, 95% CI = 1.16–1.61) and (OR = 1.36, 95% CI = 1.00–1.86), respectively (Figure 3). High heterogeneity was observed across studies (*I*² = 70.83%, p < 0.01), indicating substantial variability in the effect estimates.

**Figure 4:**
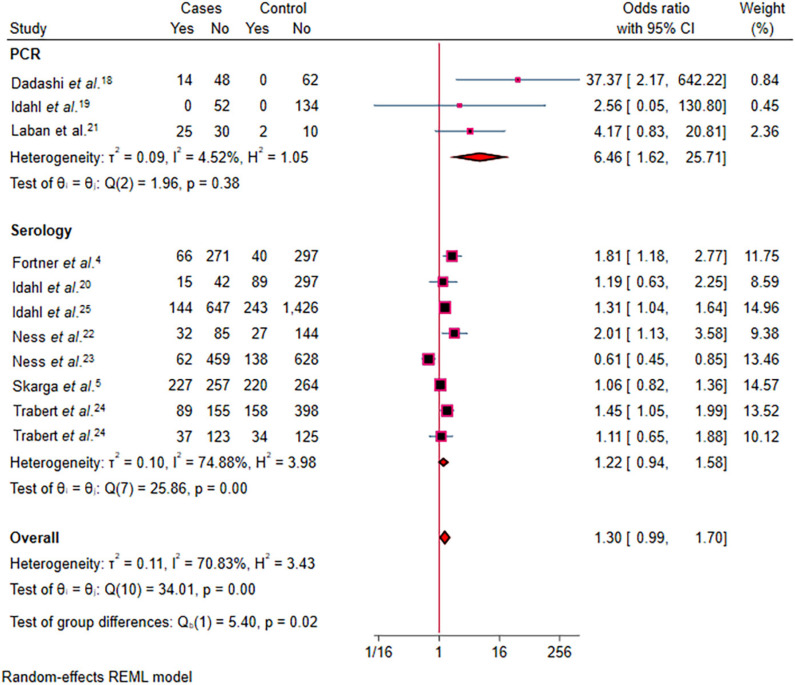
Forest plot showing the pooled random-effects analysis of the association between *Chlamydia trachomatis* infection and ovarian cancer, with OR and 95% CI presented by subgroups on diagnostic methods.

In subgroup analyses based on control group type, the association was non-significant for healthy controls (OR = 1.21, 95% CI = 0.95–1.54; Supplementary Figure S1), patients with borderline tumors (OR = 0.85, 95% CI = 0.50–1.44; Supplementary Figure S2), and patients with benign gynecological conditions (OR = 2.92, 95% CI = 0.22–38.6; Supplementary Figure S3). However, for healthy controls, the association became statistically significant when studies by Ness *et al*.^23^ and Skarga *et al*.^5^ were excluded (data not shown). Moreover, three studies using healthy controls provided adjusted OR; a random effect meta-analysis on these studies indicated that *C. trachomatis* infection is significantly associated with increased risk of OC (OR = 1.53, 95% CI = 1.15–1.95; Figure 4). In another sub-group analysis (Figure 4) based on diagnostic methods, studies that used PCR to detect *C. trachomatis* infections indicated a statistically significant association (OR = 6.64, 95% CI = 1.62–25.71), while studies that used serological assays showed non-significant association (OR = 1.22, 95% CI = 0.94–1.58). A cumulative meta-analysis showed a weakening association as more recent studies were added (Supplementary Figure S5). Funnel plots and Egger's test were employed to assess publication bias. The funnel plot for association between *C. trachomatis* infection and OC appeared asymmetrical. Moreover, Egger's test showed evidence of publication bias (β = 1.6; p-value = 0.015; Supplementary Figure S6).

## DISCUSSION

In this study, we evaluated the association between *Chlamydia trachomatis* infection and the risk of OC. The overall meta-analysis, using a random-effects model, revealed a statistically non-significant association between *C. trachomatis* infection and increased risk of OC. However, the p-value and OR were close to the significance threshold, suggesting that the association may be weak but cannot be completely ruled out. The OR of approximately 1.30 suggests a 30% increased risk of OC among women with *C. trachomatis* infection, but the CI includes 1, indicating uncertainty in the estimate. This lack of statistical significance may be partly due to variability across studies or limitations such as small sample sizes. Interestingly, sensitivity analyses that excluded studies by Ness *et al*.^23^ and Skarga *et al*.^5^ revealed a significant association, with CIs exceeding 1, indicating a more robust link between *C. trachomatis* infection and OC risk when these studies are removed. This suggests that methodological variations across studies—including the use of mixed control groups (combining benign conditions and healthy women), differences in diagnostic approaches (serology versus molecular methods), antibody targets, and adjustment for numerous covariates (including parity, smoking, and BMI)—may have contributed to conservative effect estimates, potentially explaining the inconsistent results among original studies.

A previous meta-analysis^13^ on this topic reported relatively similar estimates for the OR and CI (OR: 1.344; 95% CI: 1.19–1.50), but it found a statistically significant association between *C. trachomatis* infection and OC risk. There are several differences between that study and our current meta-analysis. Notably, we excluded two studies that lacked appropriate control groups, while the previous meta-analysis included them, which may have contributed to their stronger findings. Furthermore, our analysis incorporates more recent data, including a nested case-control study from the Finnish Maternity Cohort^5^, which reported a non-significant association between *C. trachomatis* and OC. This addition may have tempered the overall effect size in our study. These methodological differences highlight the importance of study selection criteria and the inclusion of the most up-to-date evidence when evaluating the association between *C. trachomatis* infection and OC.

Our subgroup analysis based on diagnostic methods revealed important differences in the association between *C. trachomatis* infection and OC risk depending on the diagnostic method used to detect the infection. Studies that utilized PCR^18,19,21^ for detection showed a statistically significant association (OR = 6.64), suggesting a potential link between *C. trachomatis* infection and increased OC risk. However, it is important to note that a single positive PCR result reflects an active infection at the time of testing and does not necessarily indicate chronic or persistent infection, which is more relevant to carcinogenesis. Persistent or untreated *C. trachomatis* infections over time may contribute to organ damage and tumorigenesis, rather than transient or resolved infections. Conversely, a negative PCR result at a given moment does not rule out a past infection, whose lingering effects might still contribute to oncogenesis. In contrast, studies that relied on serological assays^4,5,20,22–25^, which detect *C. trachomatis* antibodies, showed a non-significant association (OR = 1.22). While serological testing can help identify past exposure and potentially chronic infections, it lacks optimal specificity and sensitivity. These findings highlight the complexity of studying the association between *C. trachomatis* infection and OC, emphasizing the need for improved diagnostic markers to differentiate acute, chronic, and resolved infections. Further research is necessary to refine the methods used to assess infection history and to better understand the long-term impact of persistent *C. trachomatis* infection on OC development.

Although further research is required to elucidate precise pathobiological mechanisms, *C. trachomatis* may contribute to OC development through both pathological and immunological pathways^26,27^. Chronic infection with *C. trachomatis* induces persistent inflammation in the reproductive tract, particularly the fallopian tubes and ovaries^28^. This chronic inflammation leads to the release of pro-inflammatory cytokines, such as interleukin-1 (IL-1), interleukin-6 (IL-6), and tumor necrosis factor-alpha (TNF-α), which can cause tissue damage and promote cellular proliferation^28,29^. The repeated cycles of inflammation and repair may increase the likelihood of DNA damage in the epithelial cells of the ovaries, contributing to malignant transformation^30,31^. Moreover, *C. trachomatis* can evade immune detection by inhibiting apoptosis of infected cells and modifying host immune responses, leading to prolonged infection^32^. This immune evasion is further facilitated by molecular mimicry and the production of heat shock proteins, which enable the bacterium to persist intracellularly, evoking a chronic inflammatory response^33^. Additionally, *C. trachomatis* infection has been associated with dysregulated key cellular processes such as DNA repair mechanisms, oxidative stress, and epithelial-to-mesenchymal transition (EMT)^34–37^, all of which are critical in cancer development. The combined effects of these pathological and immunological disruptions create a microenvironment conducive to oncogenesis, suggesting that *C. trachomatis* may be a significant modifiable risk factor for ovarian cancer.

This study has several strengths. It provides a comprehensive and systematic review of diverse studies, enhancing the generalizability of the findings. The use of a random-effects model accounts for variability across studies, offering a more reliable pooled estimate. Further, the inclusion of sensitivity and subgroup analyses strengthens the robustness of the results. Despite the valuable insights provided by this meta-analysis, several limitations should be acknowledged. First, substantial heterogeneity was observed across the included studies, which may reflect variations in study design, populations, and diagnostic methods for *C. trachomatis* infection. This variability complicates the interpretation of the pooled effect estimates and may reduce the precision of the findings. Second, many of the studies relied on serological testing for *C. trachomatis* infection, which may not accurately capture active or chronic infections. Moreover, resolved past infections may still have caused irreversible epithelial damage, potentially contributing to carcinogenesis. The inability to precisely distinguish between past, chronic, and resolved infections remains a challenge in accurately assessing the long-term impact of *C. trachomatis* on OC risk. Moreover, relying on serology could lead to misclassification of exposure status, introducing potential bias into the results. Third, several studies had small sample sizes, limiting their statistical power to detect significant associations. Additionally, publication bias, as indicated by the funnel plot asymmetry, may have influenced the findings, with smaller studies showing null or negative results, less likely to be published. Finally, residual confounding cannot be entirely ruled out, as not all studies adjusted for important potential confounders such as sexual behavior or other sexually transmitted infections that could influence OC risk.

## CONCLUSION

In conclusion, this meta-analysis suggests a possible association between *C. trachomatis* infection and an increased risk of OC. However, the borderline statistical significance, high heterogeneity, and sensitivity of the effect size to individual studies warrant caution in interpreting the results. Future studies should prioritize: (a) standardized serial testing (combining PCR and chronicity markers like cHSP60-1 antibodies) to capture infection persistence; (b) mechanistic studies linking chronic tubal inflammation to malignant transformation; and (c) evaluation of antibiotic intervention trials on OC incidence in high-prevalence populations.
