# Supplementary material for: Chlamydia trachomatis infection and risk of ovarian cancer: a systematic review and meta-analysis
Source: Rev Inst Med Trop Sao Paulo. 2025 Jun 9;67:e34. doi: 10.1590/S1678-9946202567034 (PMC12148330; doi:10.1590/S1678-9946202567034)
Supplement: Supplementary file 1 [file 1678-9946-rimtsp-67-S1678-9946202567034-Suppl01.pdf]

REVISTA DO INSTITUTO DE MEDICINA TROPICAL DE SÃO PAULO

JOURNAL OF THE SÃO PAULO INSTITUTE OF TROPICAL MEDICINE

SUPPLEMENTARY MATERIAL

https://doi.org/10.48331/scielodata.SMWIKG

Chlamydia trachomatis infection and risk of ovarian cancer: a systematic review and meta-analysis

Pei Wang[1*], Xiuxiu You[2*], Xianjing Zeng[3], Qingmei Peng[4]

[1]Xianyang Central Hospital in Shaanxi Province, Department of Gynecology and Obstetrics, Xianyang, Popular Republic of China

[2]The Affiliated Hospital of Southwest Medical University, Department of Gynecology, Luzhou, Popular Republic of China

[3]Affiliated Hospital of Jinggangshan University, Department of General Practice Medicine, Ji'an, Popular Republic of China

[4]Jinggangshan University, Clinical School of Medicine, Department of Obstetrics and Gynecology, Ji'an, Popular Republic of China

*These authors contributed equally to the study

Correspondence to: Qingmei Peng
Jinggangshan University, Clinical School of Medicine, Department of Obstetrics and Gynecology, Ji'an, 343000, Popular Republic of China

E-mail: QingmeiPeng@outlook.com

Received: 22 January 2025

Accepted: 17 April 2025

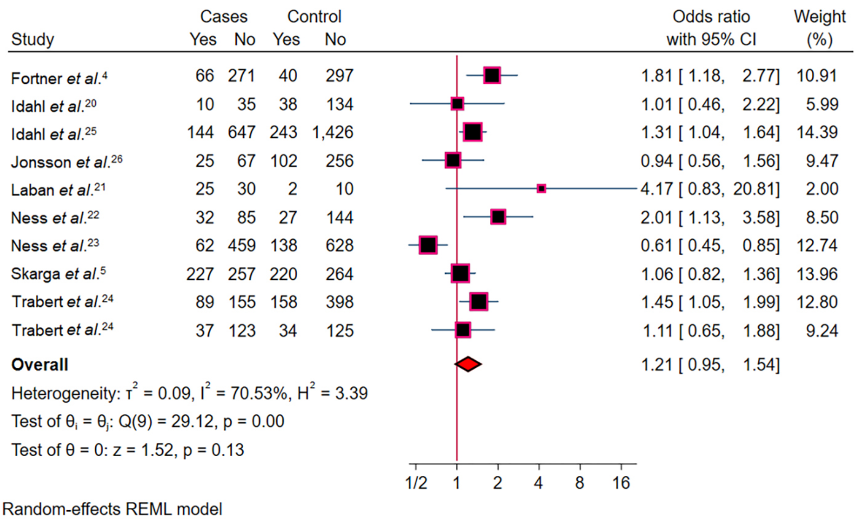

Supplementary Figure S1 - Forest plot showing the pooled random-effects analysis of the association between *Chlamydia trachomatis* infection and ovarian cancer, with odds ratios (OR) and 95% confidence intervals (CI) presented for datasets using healthy controls.

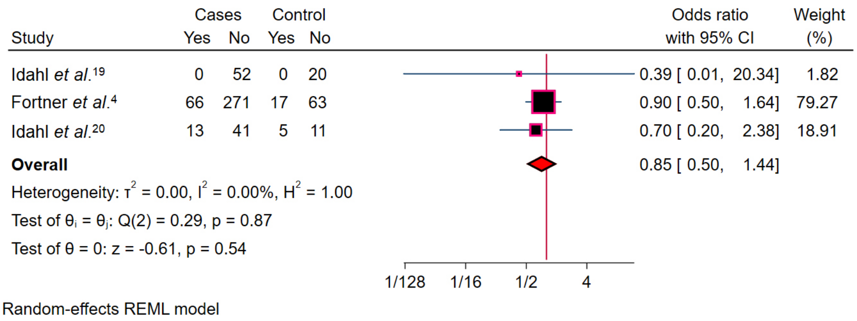

Supplementary Figure S2 - Forest plot showing the pooled random-effects analysis of the association between *Chlamydia trachomatis* infection and ovarian cancer, with odds ratios (OR) and 95% confidence intervals (CI) presented for datasets using patients with borderline tumor as controls.

This is an open-access article distributed under the terms of the Creative Commons Attribution License.

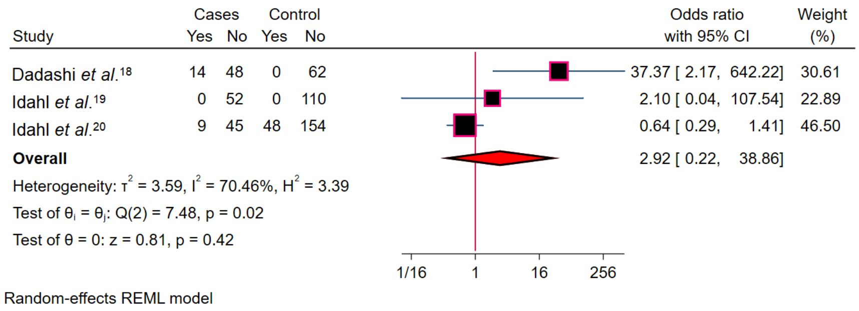

Supplementary Figure S3 - Forest plot showing the pooled random-effects analysis of the association between *Chlamydia trachomatis* infection and ovarian cancer, with odds ratios (OR) and 95% confidence intervals (CI) presented for datasets using patients with benign gynecological conditions as controls.

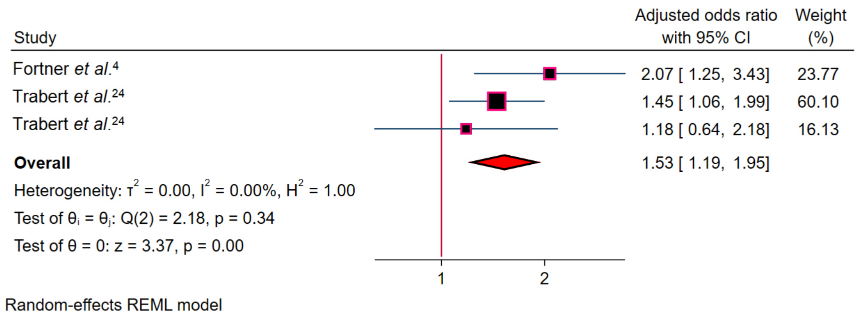

Supplementary Figure S4 - Forest plot showing the pooled random-effects analysis of the association between *Chlamydia trachomatis* infection and ovarian cancer, with odds ratios (OR) and 95% confidence intervals (CI) presented for datasets using healthy controls that provided adjusted ORs.

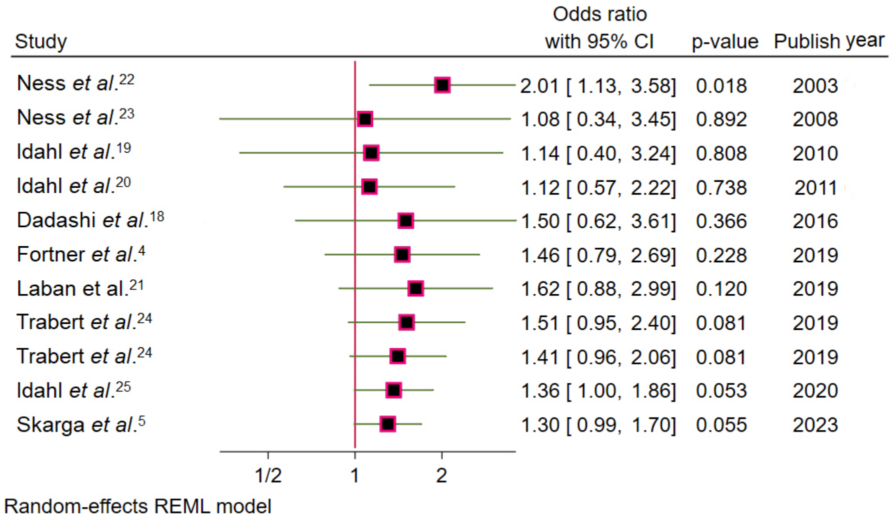

Supplementary Figure S5 - Cumulative analysis plot showing the progressive impact of individual studies on the pooled OR and 95% CI for the association between *Chlamydia trachomatis* infection and ovarian cancer. The analysis demonstrates how the overall estimate evolves as studies are sequentially added over time.

Rev Inst Med Trop São Paulo. 2025;67:e34

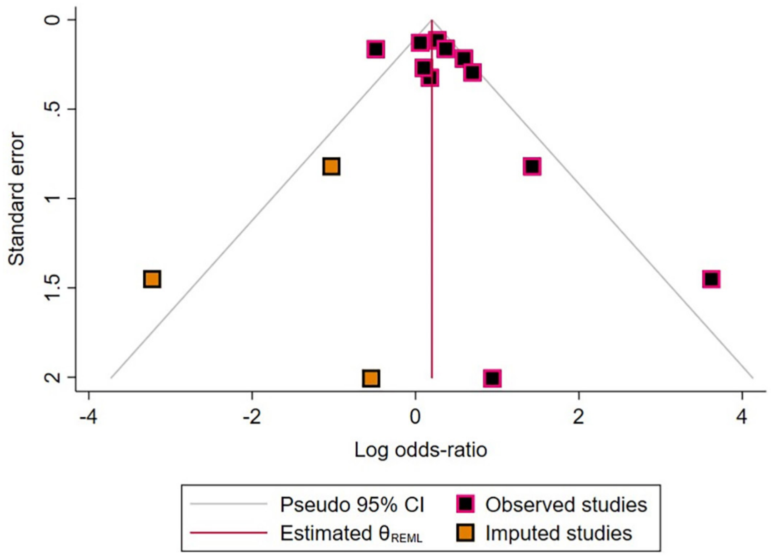

Supplementary Figure S6 - Funnel plot assessing publication bias in the studies examining the association between *Chlamydia trachomatis* infection and ovarian cancer. The symmetry of the plot indicates the absence of publication bias, suggesting that the available studies provide a balanced representation of the research findings.
